# Biomechanical modeling for the estimation of muscle forces: toward a common language in biomechanics, medical engineering, and neurosciences

**DOI:** 10.1186/s12984-023-01253-1

**Published:** 2023-09-26

**Authors:** Emilie Mathieu, Sylvain Crémoux, David Duvivier, David Amarantini, Philippe Pudlo

**Affiliations:** 1 Univ. Polytechnique Hauts-de-France, LAMIH, CNRS, UMR 8201, Campus Mont Houy, 59313 Valenciennes, France; 2 Centre de Recherche Cerveau et Cognition (CerCO), UMR CNRS 5549, Paul Sabatier University, Toulouse, France; 3 ToNIC, Toulouse NeuroImaging Center, Université de Toulouse, Inserm, Paul Sabatier University, Toulouse, France

**Keywords:** Musculoskeletal modeling, Muscle forces, Static optimization, Dynamic optimization, Forward modeling, Inverse modeling, Data tracking, Terminology homogenization

## Abstract

Different research fields, such as biomechanics, medical engineering or neurosciences take part in the development of biomechanical models allowing for the estimation of individual muscle forces involved in motor action. The heterogeneity of the terminology used to describe these models according to the research field is a source of confusion and can hamper collaboration between the different fields. This paper proposes a common language based on lexical disambiguation and a synthesis of the terms used in the literature in order to facilitate the understanding of the different elements of biomechanical modeling for force estimation, without questioning the relevance of the terms used in each field or the different model components or their interest. We suggest that the description should start with an indication of whether the muscle force estimation problem is solved following the physiological movement control (from the nervous drive to the muscle force production) or in the opposite direction. Next, the suitability of the model for force production estimation at a given time or for monitoring over time should be specified. Authors should pay particular attention to the method description used to find solutions, specifying whether this is done during or after data collection, with possible method adaptations during processing. Finally, the presence of additional data must be specified by indicating whether they are used to drive, assist, or calibrate the model. Describing and classifying models in this way will facilitate the use and application in all fields where the estimation of muscle forces is of real, direct, and concrete interest.

## Background

Musculoskeletal pathologies and neurological motor disorders are public health issues [1] in which the estimation of muscle forces in the fields of biomechanical movement analysis, medical engineering, or neurosciences can be clearly beneficial [1–3]. Accurately determining the forces exerted by each muscle acting on a joint is crucial in biomechanics to identify the mechanism behind these pathologies, improve treatments, develop new rehabilitation techniques and guide surgical procedures [2, 3]. Furthermore, determining muscle forces is also essential in neuroscience and related fields to improve the knowledge of neuro-musculoskeletal system organization and control, as well as to gain insights into origins of motor disorder [4, 5]. Finally, the estimation of muscle forces in biomedical engineering provides the necessary tools to develop movement devices (motorized prostheses, exoskeletons, ergometers) to facilitate clinical rehabilitation or assess sports performance [6–8]. Moreover, the real-time estimation of muscle forces can yield important information for human–robot interaction systems [7, 9].

Determining individual muscle forces is not an easy process. Despite the inherent limitations in modeling assumptions due to the unavoidable simplification of the extremely complex neuromusculoskeletal system, biomechanical models (more simply called “models” in this article) provide indirect access to muscle force estimation [1]. These estimations can reach an adequate level of precision and reliability for the various fields of application concerned [10, 11]. As such, there is no doubt that the estimation of the muscle forces is useful to provide accurate knowledge to adapt rehabilitation programs, prepare for surgical interventions, prevent the risks of musculoskeletal disorders, obtain indications on the origin of a motor deficit, or even design robotic prostheses. On the contrary, the terminology used to describe these models is inconsistent and seems extremely diverse between the different fields of research that develop and/or use musculoskeletal models. For example, a significant source of confusion exists regarding the description of the different steps required for the determination of the muscle forces leading some authors to speak of “forward optimization” and “inverse optimization” [6, 12]. However, these terms are only found in the field of biomechanics and have no clear meaning in other fields. This makes it difficult to understand the different model components and impedes a model’s use beyond its initial scientific field. Without questioning a model’s validity and the importance of its different components, it appears that the absence of a common language to describe biomechanical models detriments both the communication and understanding between research fields. It would be particularly relevant to establish a common language to facilitate method discussion, allow comparisons between models and discuss the advantages, disadvantages, and results. A clear description would thus avoid confusion in model comprehension and make them more accessible for practical applications. In addition, a precise and fully comprehensible description of the model would provide indications on the modeling hypotheses and simplify the choice of a model according to the desired level of estimation precision. This is a determinant point since the modeling assumptions depend on several constraints according to the considered field [3]. For example, clinicians expect very accurate results even if the data processing by the model is computer-intensive [13], while roboticists conversely favor fast solutions for real-time control [7]. Another key point is that, regardless of the desired level of precision, the estimation of muscle force requires measurable external data such as the reaction force components at the subject-environment interface, and the kinematics of body segments. However, inaccuracies during the acquisition of such data [14–16] and the use of generic anthropometric tables established from cadavers [17] can reduce estimation reliability. To provide additional information and improve the reliability of solutions obtained using modeling, additional data can be added [18]. Accurately describing, with a common language, the use of these additional data in a model is important to know their importance in the estimation process and to estimate the reliability of the results.

**Fig. 1:**
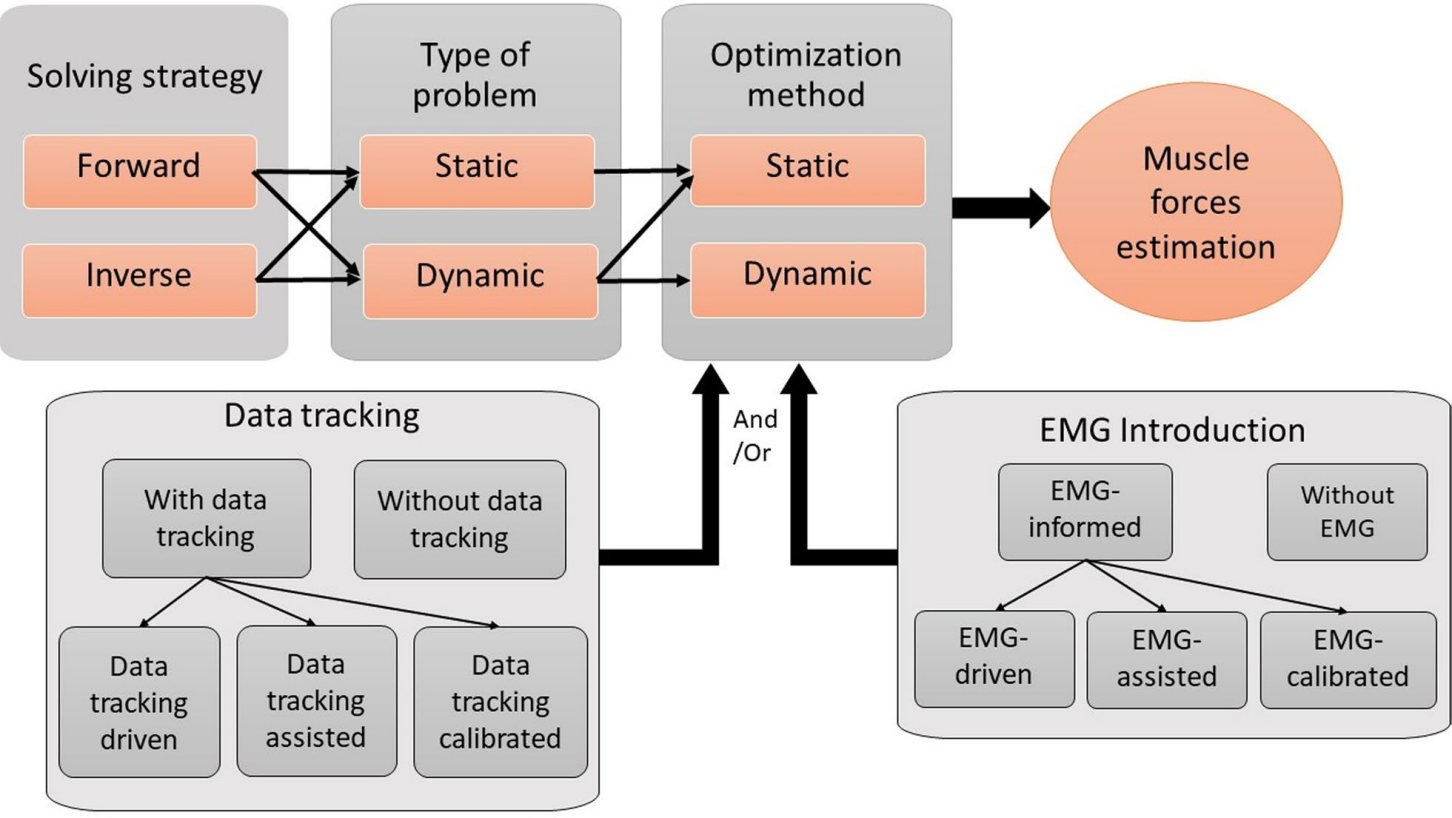
Schematic representation of different components used to describe the biomechanical models for muscle forces estimation. First, the solving strategy must be defined by specifying whether it is forward or inverse. Then, the type of problem, static or dynamic, must be described while avoiding confusion with the optimization method which can be static or dynamic. Finally, the presence of additional data must be specified by indicating whether they are used to drive, assist, or calibrate the model

Hence, the aim of the present paper is not to question the importance of muscle force estimation but to provide a form of lexical disambiguation and to propose relevant universal terminology that can be used in all fields of application, enabling the classification of existing biomechanical models (Fig. 1) using a tree structure. This paper is not a systematic review of the terminology used but a critical analysis of the terms generally used based on a literature review on musculoskeletal modeling (mainly on upper limb). To this end, we discuss the different terms used in the literature based on a basic description of the different model components and examples and we provide definitions of key terms. Firstly, we assess the strategy with which the problem is solved: forward or inverse. Secondly, we evaluate the different types of problems: static or dynamic. Thirdly, we explain, using computer science terminology, how these problems are solved via optimization processes. Fourthly, we show how additional data such as electromyography (EMG) are integrated and tracked to obtain more physiologically realistic results. Finally, we give some examples of model descriptions using the terminology we propose. Some models using neural networks or controllers, for example, may include some of the components described here but are not included in this article.

## Solving strategy

**Fig. 2:**
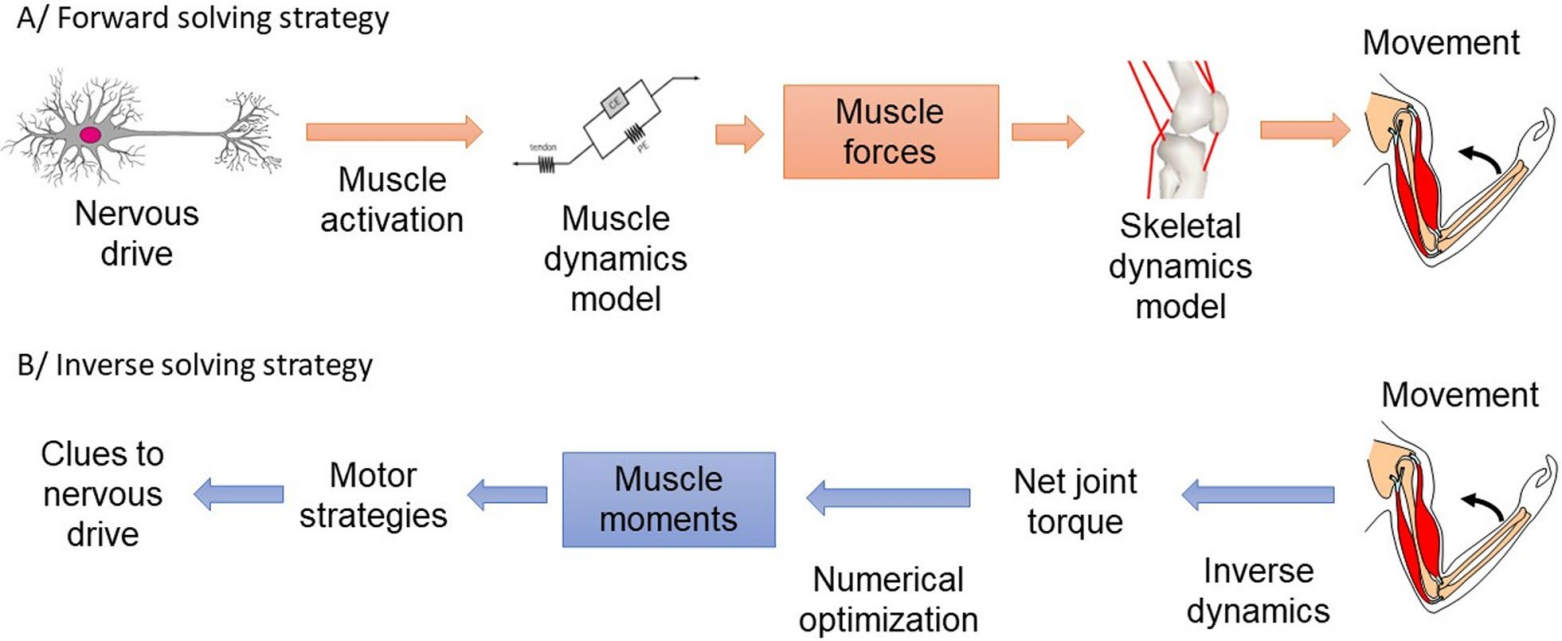
Schematic representation of models with forward (**A**) and inverse (**B**) solving strategies. In case of a forward strategy, the estimation force problem is solved starting from the nervous drive. After estimating the muscle activations, a muscle dynamics model estimates the forces produced and then possibly the movement kinematics can be reconstructed. For an inverse strategy, the muscle force estimation problem is solved starting from the movement kinematics and kinetics. The net joint torque is distributed in different muscle moments and then possibly assumptions can be made about the nervous drive

Muscle force estimation problems can be solved with two different strategies: forward or inverse (Figs. 1 and 2). With the forward solving strategy, the models aim to connect muscle activation level (i.e., level of muscle fiber recruitment) and muscle physiology to determine the force produced [1, 13]. With the inverse solving strategy, the model exploits the consequences of a muscle contraction to estimate muscle force by determining the net joint torque and dividing it into individual muscle moments [13, 14].

During voluntary muscle contractions, the neural drive modulating muscle activation is partly generated in the primary motor cortex. Neural information travels along the corticospinal tract to motor neurons and interneurons located in the spinal cord to reach the muscle fibers. The nervous system has to manage the level of muscle fiber activation of different muscles around a joint to ensure smooth movements. Buchanan et al. [14] described the activation rate of these muscle fibers, called muscle activation, as a value between 0 and 1. Electromyography (EMG), optimization, or neural network models can be used to estimate this value. With forward solving strategy, these muscle activations are the input data of models representing the physiology of the muscles and tendons with their active and passive components (usually a Hill-type model). This reflects how muscle activations are transformed into forces [6, 8, 14, 19]. Individual muscle forces operating around the joint add up to produce a net joint torque.

With inverse solving strategy, the net joint torque is determined using the kinematics of the body segments and, optionally, reaction forces. The net joint torque is then distributed among the different muscles to obtain individual muscle forces [12, 16, 20]. However, this distribution is an undetermined problem because the number of muscles involved in moving a joint is higher than the number of degrees of freedom. This problem of muscle redundancy can be solved by minimizing or maximizing an objective function, which must be chosen according to the task studied to reflect the mechanisms by which the muscles are recruited [21, 22]. Crowninshield and Brand [23] and Challis and Kerwin [16] tested different objective functions to estimate the forces produced by the muscles acting around the elbow: the weighted sum of the muscle forces, the sum of the forces normalized by the isometric maximum force, or the sum of the muscle stress. Models solved with inverse strategy are frequently used for their simplicity and speed of execution. However, this simplicity is accompanied by an inadequate consideration of the force production physiology and a lack of reliability in the results.

While this distinction between the terms “forward” and “inverse” allows a clear description of the solving strategy, other authors such as Anderson and Pandy [24] combined inverse and forward solving strategies with static and dynamic optimization, respectively (Table 1). This link implies that the optimization method is determined by the solving strategy and suggests that the inverse strategy is static, and the forward strategy is dynamic (a comprehensive description of static and dynamic problems is given in the following section). This link between the solving strategy, the type of problem, and the optimization method may be suitable in some fields but can be a source of confusion and model misunderstanding. The type of problem (further outlined below) and the optimization method for muscle force estimation can be static or dynamic regardless of the solving strategy (Fig. 1). Hence, the first element of the model description should be the solving strategy: forward or inverse, as defined in Table 2 (see: “Forward solving strategy” and “Inverse solving strategy”, respectively).

**Table 1:** Example of classification of some biomechanical models for muscle forces estimation

	Description by authors	Solving strategy	Type of problem	Optimization method		Additional information	
				Type of optimization	Time windows resolution	EMG contribution	Data tracking
[2]	EMG-informed	Forward	Dynamic	Static	Continuous	EMG-driven	Data tracking calibrated (Net joint torque, joint contact forces)
[7]	Inverse dynamics, Neural networks	Inverse	Dynamic	Static	Discrete	Without	Without
[14]	Forward dynamic, calibrated, EMG-based	Forward	Dynamic	Static	Continuous	EMG-driven	Data tracking calibrated (Net joint torque)
[16]	Inverse dynamics	Inverse	Static	Static	Discrete	Without	Without
[18]	Hybrid model (forward and inverse) Static optimization EMG-informed	Forward	Dynamic	Static	Discrete	EMG-driven	Data tracking assisted (Net joint torque, muscle excitations)
[35]		Inverse	Static	Static	Discrete	Without	Without
[36]	Dynamic optimization Data tracking problem	Forward	Dynamic	Static	Continuous	Without	Data tracking driven (Ground reaction forces)
[38]	Inverse dynamic, numerical optimization, EMG-assisted	Inverse	Dynamic	Static	Continuous	EMG-assisted	Data tracking calibrated (Net joint torque)
[41]	tracking-assisted forward-dynamic optimizations	Forward	Dynamic	Static	Continuous	Various models are presented	Data tracking driven
[46]	Inverse dynamics	Inverse	Static	Static	Discrete	Without	Without
[47]	Kinematics and EMG based	Inverse	Dynamic	Static	Discrete	EMG-calibrated	Data tracking driven (Net joint torque)
[48]	EMG-assisted optimization	Inverse	Dynamic	Static	Continuous	EMG-driven	Without
[51]	Forward dynamic, tracking simulation	Forward	Dynamic	Static	Continuous	EMG-driven	Data tracking calibrated (Joint angle)
[52]	inverse dynamic, Kinematic tracking	Inverse	Dynamic	Static	Discrete	Without	Data tracking assisted (Joint angle and velocity)
[21]	Inverse dynamics, Static optimization	Inverse	Static	Static	Discrete	Without	Without
[50]	Numerical optimization, EMG driven	Forward	Dynamic	Static	Continuous	EMG-driven	Data tracking calibrated (Net joint torque)
[6]	Inverse and forward optimization, EMG driven	Forward	Dynamic	Static (× 2)	Continuous and discrete	EMG-driven	Data tracking assisted (Activation)
[19]	Forward dynamic, Static optimization, EMG driven	Forward	Dynamic	Static	Continuous	EMG-driven	Data tracking calibrated (Net joint torque)
[53]	EMG driven	Forward	Dynamic	Static	Continuous	EMG-driven	Data tracking calibrated (Net joint torque)
[17]	Forward dynamics, EMG driven	Forward	Dynamic	Static	Continuous	EMG-driven	Data tracking calibrated (Net joint torque and joint angle)
[26]	EMG driven	Forward	Dynamic	Static	Continuous	EMG-driven	Data tracking calibrated (Net joint torque)
[12]	Inverse-dynamics optimization and inverse-forward-dynamics models	Inverse	Dynamic	Static	Discrete	Without	Data tracking assisted (Joint angle and velocity)
[8]	EMG driven	Forward	Dynamic	Static	Continuous	EMG-driven	Data tracking calibrated (Joint angle)
[42]	Calibrated, EMG-informed	Forward	Dynamic	Static	Continuous	EMG-driven	Data tracking calibrated and assisted (Net joint torque)
[20]	Inverse dynamics	Inverse	Dynamic	Static	Discrete	Without	Without

Proposal of a new classification of references presenting a model for the estimation of muscle forces available in biomechanics and associated fields, based on 4 superimposed components (solving strategy, type of problem, optimization method, additional information) and using the semantic elements of the common language recommended to facilitate the use, development, and applications of biomechanical modeling in all the fields where the estimation of muscle forces is of direct interest

**Table 2:** Definitions of principal terms

Term	Definition
Muscle force estimation problem	Problem consisting in seeking a muscle effort estimation [1]. Muscle efforts are related to muscle force or muscle moment production
Solving strategy	Strategy chosen to solve the muscle force estimation problem which may or may not correspond to the physiological motor control
Forward solving strategy	Strategy chosen to solve the muscle force estimation problem which corresponds to the physiological motor control, i.e., from nervous drive to force production
Inverse solving strategy	Strategy chosen to solve the muscle force estimation problem which does not correspond to the physiological motor control, i.e., from the movement kinematics and external forces to muscle moment production
Muscle excitation	Nervous drive controlling muscle contraction [14]
Muscle activation	Rate of motor units’ recruitment within the muscle [14]
Type of problem	Designates the static or dynamic aspect of the solution sought to the muscle force estimation problem. In case where a single value of muscle force at a given time t is sought, the problem is a static type. Conversely, if a change of muscle force produced over time is sought, the problem is a dynamic type
Optimization method	Mathematical method used to solve the muscle force estimation problem
Time windows resolution	Designates the temporal aspect of optimization implementation. A discrete-time optimization searches for a solution for each temporal node individually. For continuous-time optimization, a solution is sought over the entire duration of the experiment in a single step
Static optimization	Process that allows an optimal solution to be found to a problem for which all the input data are known and can be processed independently of the time course [27]
Dynamic optimization	Process that allows an optimal solution to be found to a problem for which data being acquired and can’t be processed independently of the time course [28]
Experimental situation of interest	Period during which the acquired data correspond to the situation to which the study problem relates
Data tracking	Process that allows the tracking of experimental data to ensure the realism of muscle force estimation. Estimations are compared and adjusted to experimental data [1]

## Type of problem

Depending on the task, muscle forces can be estimated by a single value at a single time point for a static problem; otherwise, for a dynamic problem, muscle force variation can be followed over time. Dul et al. [25] studied the forces produced under isometric conditions by the knee flexor muscles with an experimental protocol including isometric contractions at different intensities. This static problem consisted of calculating the muscle forces produced at a given time according to contraction intensity. Conversely, Lloyd and Besier [26] developed a model to obtain the forces produced by the muscles surrounding the knee during different tasks (eccentric flexion/extension, concentric flexion/extension, straight running, crossover cut). In their dynamic problem, the forces were estimated over the duration of the task. While there is no point in solving static problems using a dynamic optimization, dynamic problems can involve either static or dynamic optimization.

The type of problem is not usually explicitly described in the articles on muscle force estimation (Table 1), even methodological articles. Referring to the arguments developed above and to the definition given in Table 2 (see: “Type of problem”), we recommend that the type (i.e., “static” or “dynamic”) of problem be clearly stated in the model description to avoid confusion with the optimization method.

## Optimization method

The optimization method can involve static or dynamic optimization (Fig. 1). According to computer science, static optimization can be defined as a process that allows an optimal solution to be found for a problem in which all the input data are known and can be processed independently of the time course [27]. We define “optimal solution” as at least one *near-optimal* solution (possibly several) found in a given amount of time. Dynamic optimization problems are special cases of dynamic problems that “are solved online by an optimization algorithm as time goes by” [28]. While the set of possible solutions is defined before static optimization, it can be modified during dynamic optimization [29–31]. This search for optimal solutions can be carried out in different resolution time windows: if the problem is solved at each time interval, the optimization is in discrete time; if the problem is solved for the entire duration of the task studied in a single step, the optimization is in continuous time [32].

In practice, static optimization is used in static or dynamic problems to calibrate or drive models with forward solving strategy and to solve the problem of muscle redundancy with inverse solving strategy [1, 13, 24, 33]. Many parameters are needed to estimate individual muscle forces using the forward solving strategy. These parameters can be the maximal force, optimal length, maximal shortening velocity, the force–length and force–velocity relationships, and the parameters allowing for muscle activation estimation. The latter values can be obtained from measurements made on cadavers [34] but these parameters vary from one individual to another and depending on the task [17]. A calibration process is usually performed before experimental situation of interest, as the reliability of muscle force estimation depends on the individualization of these parameters [35]. For example, Hou et al. [19] used a set of data (joint angles and EMG) acquired before the experimental situation of interest to estimate muscle activation and the muscle parameters of their model with forward solving strategy. A static optimization process determined the optimal values of these parameters to minimize the difference between the net joint torque estimated by the model and the same values determined through experimental data. Likewise, Lin and Pandy [36] used static optimization to find a set of muscle excitations to drive a model using OpenSim software [37]. They collected gait data and calculated the set of muscle excitations that reproduced the experimental data over one stride cycle, minimizing the difference between the estimated and measured ground reaction forces. These two optimizations conducted by [19] and [36] in a model with forward solving strategy were performed in one step for all the data from the experimental situation of interest (the time is not divided into time intervals, it is a continuous variable) and so are continuous-time static optimizations [32]. This type of optimization is often mistakenly regarded as dynamic optimization due to the continuity over time of the result obtained (Table 1). Models with inverse solving strategy can also be associated with continuous-time static optimization. As an example, Amarantini et al. [38] estimated agonist and antagonist muscle moments with coefficients applied to EMG data, and used a static optimization process in one step for the whole session data to estimate the values of these coefficients.

Static optimization problems can also be solved at each time interval [1, 13, 24, 33, 36]. For example, Challis and Kerwin [16] distributed muscle forces using static optimization to solve the problem at each time point without correlation with the results from the previous or next instant in time. However, this type of optimization can lead to discontinuities in the results obtained over time. In this study, time is an integer parameter, and the time interval is fixed. They solved a static optimization problem in discrete time [32].

Although it seems unnecessary to specify the resolution time windows (as defined in Table 2) in model descriptions, it is essential to avoid any confusion between the type of problem and the optimization method (as both can be static or dynamic) and not systematically link the continuous aspect with dynamic optimization.

To our knowledge, there is no muscle force estimation model that uses a dynamic optimization process as defined above. Therefore, we will refer to the field of computer science to provide an example of dynamic optimization. Several variants of the well-known dynamic path planning problem are good candidates to test static *vs.* dynamic optimization processes [31, 39, 40]. Even if the problem considered is based on routing helicopters in three dimensions, this is quite similar to a classical Global Positioning System (GPS). The objective is to determine an optimal route (or at least a sub-optimal route) to go from point S (start) to point D (destination) that minimizes the distance and/or time and/or fuel consumption while fulfilling constraints related to the vehicle and so on. Before the trip, considering the conditions as ideal, a route is obtained using static optimization. During the trip, the route is dynamically re-optimized to deal with real-time problems (traffic jams, meteorological events, breakdowns, addition/deletion of waypoints…). Optimization is therefore carried out in a changing environment and must adapt to changes made in real time. Muscle force estimation models may involve dynamic problems but have not yet been used in changing environments requiring adaptation of the objective function or constraints over time.

For a better understanding of the model process and characteristics used to estimate muscle force, it seems necessary to specify whether the optimization method is static or dynamic (Fig. 1) as defined in Table 2 (see: “Static optimization” and “Dynamic optimization”, respectively). Regardless of the method, optimization provides an optimal result that does not necessarily correspond to the experimental data, and which is not systematically physiologically realistic.

Beyond the needful clarifications regarding the semantic elements relating to the solving strategy, and some characteristics of optimization problems designed to estimate muscle forces, further clarifications seem necessary regarding the possibility of adding additional information from experimental data.

## Additional information

There seems to be a consensus on the usefulness of additional information to improve the reliability of the results obtained with biomechanical models [18, 41, 42]. EMG data reflect the sum of the action potentials received by muscle motor units and provide information on how the nervous system controls muscle contractions. These data are important to improve the accuracy of the estimated muscle forces. Moreover, new approaches characterizing how the nervous system drives muscle coordination constitutes a field of research that deserves to be investigated to provide a more realistic and neurophysiologically-compatible estimation of muscle forces [18, 42]. Other experimental data can be considered to ensure a good correlation between the experimental data and the solutions provided by the model. However, the terminology used to describe their integration into models is heterogeneous and requires clarification.

### Contribution of EMG

EMG data reflect the central neural drive from the cortex to the muscle and can be used with forward or inverse solving strategies (Fig. 1). Models using EMG data are called EMG-informed models [2, 42] and are further designated by a variety of sub-terms such as EMG-driven, EMG-assisted, EMG-hybrid [2], EMG-based [38], and EMG–force [43] (Table 1). These last two terms (EMG-based and EMG–force) are not widely used in the literature and the term EMG-hybrid does not refer directly to the EMG implementation process. To clarify this terminology, the classification proposed by Hoang et al. [2] can be simplified to present only two categories: “EMG-driven” in which EMG is used as an essential input to drive the model, and “EMG-assisted” in which EMG provides additional information to improve solution reliability but its removal would not hinder the estimation of muscle forces. However, this classification has to be expanded to take into account “EMG-calibrated” models in which EMG helps to calibrate the model before applying it to the experimental situation of interest.

Buchanan et al. [14] proposed to transform the EMG signals into muscle activations to drive a model with forward solving strategy. For this purpose, the EMG signals were normalized by their maximal value during a maximal voluntary contraction and filtered to obtain muscle excitations. These muscle excitations were transformed into neural activations to take into account the electromechanical delays (i.e., the delay between neural drive and force production) [6, 14, 44]. According to their study, this transformation was achieved with a differential equation or a recursive filter. The neural activations were then adjusted to consider the non-linear aspect of the EMG–force relationship [6, 14, 45]. In this context, the EMG signals are input data *driving* these models, which can be called EMG-driven models [1–3, 14, 26, 42].

With the inverse solving strategy, EMG provides additional information to divide the net joint torque into individual muscle moments to obtain more realistic results. The objective functions tested by Crowninshield and Brand [23] or Challis and Kerwin [16], are commonly used but they can fail to correctly predict the co-contraction of antagonist muscles [46, 47]. The minimization of muscle forces or muscle stresses leads to an underestimation of the forces acting in the opposite direction of the movement. To improve the estimation of antagonist muscle forces, EMG data can be added to guide the force distribution during optimization. Amarantini et al. [38] minimized the difference between the net joint torque determined by inverse dynamics and the net joint torque estimated through an EMG-torque relationship obtained with a static optimization. This optimization determines the values of the coefficients representing the EMG-torque relationship, individual muscle gains, stiffness, and viscosity that were applied to the EMG data to estimate net agonist and antagonist torques. These muscle group torques were then divided into individual muscle moments using a min/max optimization process with an objective function based on muscle stress. This is a typical EMG-assisted model [48] as EMG data were used as additional data to limit the set of possible solutions of the optimization process.

The EMG data can also help to calibrate the objective function of the optimization process. Wen et al., [47] employed a model with a forward solving strategy using EMG, and an inverse solving strategy with an objective function including a co-contraction factor, *Xs*, at an initial value. These two estimations of muscle forces, with forward and inverse strategies, were compared, and *Xs* was adjusted to minimize the difference between them. This process was applied to data from 17 subjects to find a relationship between *Xs* and kinematic data using linear regression. Once this calibration was performed, it was possible to estimate the muscle forces without EMG data. This is an example of a model that can be called EMG-calibrated because EMG data allow the objective function to be calibrated but are not useful during the experimental situation of interest.

### Data tracking

The term “data tracking” refers to a process during which an optimization or a controller minimizes the difference between the data estimated using the model and the experimental data. The tracking data can be joint torques [19, 49, 50], kinematics [51], or muscle activations [6] (Table 1). The tracking information allows for more realistic results to be obtained, and limits inter-trial variability and sensitivity to noise. Data tracking can be conducted before model application to the experimental situation of interest during a calibration process, or directly during the experimental situation of interest. In the latter case, the terminology used to describe how data tracking contributes to the estimation of forces varies. For example, “enhanced static optimization” [3], “closed-loop control strategies” [52], “inverse forward optimization” [12], “assisted data tracking” [1, 13], and “tracking methods” [51] all refer to a form of data tracking process. Some of these terms only apply to a minority of existing models because they are too specific to a model configuration. For example, the term “enhanced static optimization” could only apply in the case of static optimization. In addition, we eliminated terms that did not directly refer to the process described. The terms “closed-loop control strategies” and “inverse forward optimization” do not directly refer to experimental data tracking. Finally, some terms are too generic, such as “tracking methods”, and impede the differentiation of different possible configurations. To clarify this terminology, we propose to follow the same reasoning as with EMG contribution, and can define three categories (Fig. 1): “data tracking-driven” models in which data tracking is used as an essential input to drive the model; “data tracking-assisted” models where data tracking brings additional information to improve solution reliability, but its removal would not hinder the estimation of muscle forces; and “data tracking-calibrated” models in which data tracking helps to calibrate the model before its use in experimental situation of interest.

Data tracking-driven processes can be used to drive models with forward solving strategy. Lin and Pandy, [36] tracked external forces to determine a set of muscle activations that correspond to experimental data to drive their model. This approach, providing solutions that reflect the dynamics of the system, is often assimilated to dynamic optimization [24]. However, even if the force estimation problem is dynamic, the set of possible solutions does not change during the optimization; the environment remains stable. This dynamic problem is therefore solved using continuous-time static optimization (Table 1).

Data tracking-assisted processes can be used to enhance the reliability of the estimated forces in an additional feedback loop that can be removed. For example, data tracking can limit errors linked to net joint torque computation using inverse dynamics and allow for better consideration of muscle physiology with inverse solving strategy. Rengifo et al. [52] determined the joint torque with inverse dynamics and then divided it into individual muscle forces using an objective function with a co-contraction factor. The corresponding kinematics were computed using a geometrical musculoskeletal model; a controller minimized the difference between the estimated and experimental kinematics to adjust the net joint torque.

Finally, data tracking-calibrated processes can aid model adjustment for the subject and the task before the experimental situation of interest. Koo and Mak [53] applied a model with forward solving strategy to estimate the forces produced at the elbow. To calibrate their model, the subjects carried out maximal isometric contractions and muscle parameters were optimized by tracking the maximal isometric torques.

In summary, in Table 1, we present a classification with which each of the models cited as an example would be characterized following the common language that we propose.

## Conclusion

As with semantic clarifications that have already been proposed on other topics [54–56], the goal of this paper is to provide a common language to define, report, and classify biomechanical models for the estimation of muscle forces and moments. We do not question the relevance of the models or the terminology, which is generally correct in each field. The idea is to encourage researchers to consider the proposed semantic adjustments to make the description of the components of their model easier to understand, communicate, and interpret. It is also a question of resolving some issues regarding the apparent contradiction between articles and avoiding misunderstandings relating to how the models are conceived. Ultimately, this will facilitate the use and applications in various fields where the estimation of muscle forces is of real, direct, and concrete interest.

First, the solving strategy must be defined by specifying whether it is forward or inverse. Then, the type of problem, static or dynamic, must be described while avoiding confusion with the optimization method which can be static or dynamic. Finally, the presence of additional data must be specified by indicating whether they are used to drive, assist, or calibrate the model.

These semantic recommendations are based on a synthesis of terms used in the main fields that develop or use models to estimate muscle forces. This synthesis was carried out in way that the selected terms can be adapted for most of the existing models and by excluding the terms specific to the configuration of few models or too broad. Ultimately, we propose a common language for which the systematic use seems particularly justified in all these fields in order to facilitate understanding of the use, development, and solutions of models for engineers, clinicians, and researchers in all related fields. As a future perspective, to further validate this new terminology, a questionnaire could be sent to the scientific community using or developing musculoskeletal models to compare it with their practices and determine its acceptance.

## Data Availability

Not applicable.
